# Acquisition of cancer stem cell properties in osteosarcoma cells by defined factors

**DOI:** 10.1186/s13287-020-01944-9

**Published:** 2020-10-02

**Authors:** Shuichi Fujiwara, Teruya Kawamoto, Yohei Kawakami, Yasufumi Koterazawa, Hitomi Hara, Toshiyuki Takemori, Kazumichi Kitayama, Shunsuke Yahiro, Kenichiro Kakutani, Tomoyuki Matsumoto, Takehiko Matsushita, Takahiro Niikura, Michiyo Koyanagi-Aoi, Takashi Aoi, Ryosuke Kuroda, Toshihiro Akisue

**Affiliations:** 1 Department of Orthopaedic Surgery, Kobe University Graduate School of Medicine, 7-5-1 Kusunoki-cho, Chuo-ku, Kobe, 650-0017 Japan; 2 Division of Orthopaedic Surgery, Kobe University Hospital International Clinical Cancer Research Center, Kobe, Japan; 3 Department of iPS Cell Applications, Kobe University Graduate School of Medicine, Kobe, Japan; 4 Division of Advanced Medical Science, Graduate School of Science, Technology and Innovation, Kobe University, Kobe, Japan; 5 Division of Gastrointestinal Surgery, Kobe University Graduate School of Medicine, Kobe, Japan; 6 Center for Human Resource development for Regenerative Medicine, Kobe University Hospital, Kobe, Japan; 7 Department of Rehabilitation Science, Kobe University Graduate School of Health Sciences, Kobe, Japan

**Keywords:** Cancer stem cells, Chemoresistance, Osteosarcoma, Sarcosphere, Tumorigenicity

## Abstract

**Background:**

Cancer stem cells (CSCs) are considered to be responsible for tumor initiation, formation, and poor prognosis of cancer patients. However, the rarity of CSCs in clinical samples makes it difficult to elucidate characteristics of CSCs, especially in osteosarcoma (OS). The aim of this study is to verify whether it is possible to generate CSC-like cells by transducing defined factors into an OS cell line.

**Methods:**

We retrovirally transduced the Octamer-binding transcription factor 3/4 (*OCT3/4*), Kruppel-like factor 4 (*KLF4*), and SRY-box transcription factor 2 (*SOX2*) genes into the MG-63 human OS cell line (MG-OKS). Parental and GFP-transduced MG-63 cells were used as negative control. We assessed the properties of the generated cells in vitro and in vivo. Multiple comparisons among groups were made using a one-way analysis of variance (ANOVA) followed by post hoc testing with Tukey’s procedure.

**Results:**

MG-OKS cells in vitro exhibited the significantly increased mRNA expression levels of CSC markers (*CD24*, *CD26*, and *CD133*), decreased cell growth, increased chemoresistance and cell migration, and enhanced sphere formation. Notably, MG-OKS cells cultured under osteogenic differentiation conditions showed strongly positive staining for both Alizarin Red S and alkaline phosphatase, indicating osteogenesis of the cells. Gene ontology analysis of microarray data revealed significant upregulation of epidermal-related genes. Tumors derived from MG-OKS cells in vivo were significantly larger than those from other cells in μCT analysis, and immunohistochemical staining showed that Ki-67, osteocalcin, and HIF-1α-positive cells were more frequently detected in the MG-OKS-derived tumors.

**Conclusions:**

In this study, we successfully generated OS CSC-like cells with significantly enhanced CSC properties following transduction of defined factors.

## Introduction

Osteosarcoma (OS) is the most common type of primary malignant bone tumor in children and young adults. OS arises predominantly in the long bones, especially the distal femur, followed by the proximal tibia, and the proximal humerus, with lungs being the most common site of metastasis of the disease [1]. Although the progress of neoadjuvant and adjuvant chemotherapies along with limb-sparing surgery has improved both the prognosis of patients with OS and their limb function, the 5-year survival rate has plateaued at around 70% for patients with non-metastatic lesions, and the rate for the patients with metastatic or recurrent or chemotherapy-resistant lesions has remained below 20% [2, 3]. Therefore, it is important to elucidate the molecular mechanisms underlying the progression, chemoresistance, and metastasis of OS [4].

To a certain extent, the poor prognosis of patients with OS could be ascribed to the chemoresistance to treatment. Chemoresistance in OS appears to be associated with various mechanisms, including removal of intracellular drug accumulation [5], enhanced DNA repair [6], perturbations in signal transduction pathways [7], autophagy-related chemoresistance [8], and microRNA (miRNA) dysregulation [9]. Recent studies have suggested that cancer stem cells (CSCs) should play crucial roles in cancer progression and relate to the chemoresistance and metastatic capabilities of cancers [10]. However, it is known that CSCs constitute only a small percentage of tumor cells; therefore, targeting CSCs for treatment or for research, especially in rare cancers, such as OS, presents many challenges.

In previous reports, several methods have been developed to isolate subpopulations with enriched stem cell properties from OS [11, 12]. However, as CSCs comprise only a small population in cancer tissues, sampling limitations remain a major obstacle in the research of CSCs. To this day, an effective (quantitatively and qualitatively) isolation method for CSCs from OS has not been established.

To overcome this challenge, we previously developed a novel method to generate CSC-like cells. We retrovirally transduced a set of defined factors, Octamer-binding transcription factor 3/4 (*OCT3/4*), Kruppel-like factor 4 (*KLF4*), and SRY-box transcription factor 2 (*SOX2*) into human colon [13] and lung [14] cancer cells, followed by culturing with a conventional serum-containing medium, but not a human embryonic stem cell medium. Cancer cells transduced with the three factors showed significantly enhanced CSC properties in terms of the expression of marker genes, formation of spheres, chemoresistance, and in vivo tumorigenicity, and were capable of forming tumors that were similar to human colon and lung cancer tissues in terms of both their structural and immunohistological patterns [13, 14]. Moreover, we identified glycogen synthase kinase 3 beta (GSK3B) and interleukin-6 (IL-6) as novel potential therapeutic targets in colon and lung cancers, respectively, using organoids, 3D structures derived from the CSC-like cells [13, 14]. However, this technique has not previously been applied in OS cells, and many aspects of the mechanism remain unexplained.

In the current study, we transduced the three factors into OS cells, resulting in the induction of CSC properties, in terms of the elevated expression of CSC markers, slow cell proliferation, higher chemoresistance, enhanced sphere formation, increased migration and osteogenic ability in vitro, and enhanced tumorigenicity in vivo. Moreover, we performed microarray analysis and identified the differentially expressed genes between the OS CSC-like cells and the control cells, with the goal of identifying the principal genes associated with the molecular mechanisms of progression, metastasis, and chemoresistance, as well as revealing novel effective therapeutic targets in human OS.

## Methods

### Cells

The human OS cell lines (MG-63 and NOS-1) were used in this study (RIKEN BRC through the National Bio-Resource Project of the MEXT, Ibaraki, Japan). Cells were cultured in Dulbecco’s modified Eagle medium (Sigma-Aldrich, St Louis, MO, USA) supplemented with 10% fetal bovine serum (FBS; Sigma-Aldrich), 100 U/mL penicillin (Sigma-Aldrich), and 100 μg/mL streptomycin (Sigma-Aldrich) and were maintained in a humidified atmosphere with 5% CO_2_ at 37 °C. Plat-A packaging cells (Cosmo Bio Co., LTD, Tokyo, Japan) were used for the production of the retrovirus. In Plat-A cultures, 1 mg/mL of puromycin (Life Technologies, Grand Island, NY, USA) and 10 mg/mL of blasticidin (Life Technologies) were added.

### Retroviral transduction

For gene transduction, we employed a modified version of the retroviral infection method previously described [15, 16]. We used the polycistronic retroviral vector designed to encode *OCT3/4*, *KLF4*, and *SOX2* in the pMX retroviral vector (pMX-OKS) (Supplemental Fig. S1A). Concomitantly, pMX with GFP (pMX-GFP) was used as a control vector. To produce retroviral particles, Plat-A cells (in DMEM with 10% FBS without antibiotics) were transfected with the retroviral vector (pMX-OKS or pMX-GFP) using the FuGene HD transfection reagent (Promega, Madison, WI, USA) following the manufacturer’s instructions. The medium was replaced at 24 h after transduction, and the retrovirus-containing supernatant was harvested at 48 h after transduction. The supernatant was filtered through a 0.45-μm pore-size syringe filter (Sartorius Stedim Biotech, Goettingen, Germany). Infection of both cell lines with the retrovirus was conducted in the presence of 4 μg/mL polybrene (Nacalai Tesque, Kyoto, Japan) for 24 h. Non-transfected cells (MG-parental and NOS-parental), as well as cells transfected with GFP (MG-GFP and NOS-GFP), were used as control. All gene transduction procedures were performed in accordance with the National Institutes of Health Guidelines, and the study protocol was approved by the Kobe University Institutional Committee (Permission no. 30-18).

### RNA isolation and real-time quantitative reverse-transcription polymerase chain reaction (RT-qPCR)

Total RNA was extracted from cultured cells and tumor tissues using an RNeasy mini kit (Qiagen, Valencia, CA, USA), and qPCR reactions were performed with the SYBR Green master mix reagent (Applied Biosystems, Foster City, CA, USA) on the ABI prism 7500 sequence-detection system (Applied Biosystems) according to the manufacturer’s instructions. Relative mRNA expressions of transduced genes (*OCT3/4*, *KLF4*, and *SOX2*), previously reported markers (*CD24*, *CD26*, and *CD133*), chemoresistance-related gene (*ABCB1*), and osteogenic differentiation-related genes (*osteocalcin*, *BMP2*, *BMP4*, and *BMP6*) were assessed. The details are given in the Supplemental Data.

### Cell proliferation and cell migration assay

We assessed cell proliferative activities by performing cell counting using a hemocytometer for measurement of total cell count and WST-8 assays using a colorimetric Cell Counting Kit-8 (CCK-8; Dojindo Inc., Kumamoto, Japan), whereby a formazan dye color intensity is directly proportional to the viable number of cells. The migration ability of cells was evaluated using a scratch wound healing assay [17]. The details are given in the Supplemental Data.

### Analysis of chemoresistance to doxorubicin

To assess the chemoresistance to doxorubicin (DOX) in cells with and without gene transduction, the viability of cells under exposure to DOX was measured by performing the WST-8 assays using CCK-8 (Dojindo Inc.). A total of 5 × 10^3^ cells at 10 days after retroviral transduction were seeded in 96-well plates, and after 24 h, the medium was replaced with DMEM containing 0 (as control), 0.3, or 30 μM of DOX (Sigma-Aldrich). The WST-8 assays were performed after 48 h of incubation, and the relative viability of cells was calculated. The expression level of the ATP-binding cassette subfamily B member 1 (*ABCB1*; also known as *MDR1*) gene in the DOX-treated cells was also examined by qPCR analysis.

### Induction of osteogenic differentiation and evaluation of osteogenic ability in vitro

Osteogenic differentiation was evaluated by alkaline phosphatase (ALP) staining and by assessment of calcium in deposited minerals using Alizarin Red S staining. A total of 1 × 10^5^ cells at 7 days after retroviral transduction were seeded in 24-well plates, following which, the medium was replaced with the osteogenesis culture kit (Cosmo Bio). After 3 weeks of incubation, staining of ALP and Alizarin Red S was performed using an ALP staining kit (Cosmo Bio) and a Calcified Nodule Staining kit (Cosmo Bio) following the manufacturer’s instructions, respectively. Concomitantly, the mRNA expressions of *osteocalcin* and bone morphogenetic protein (BMP) family members (*BMP2*, *BMP4*, and *BMP6*), which stimulate osteoblast differentiation [18, 19], were also assessed in the cells by qPCR analysis.

### Sphere formation assay

Cells (1 × 10^5^ cells/well) at 10 days after retroviral transduction were seeded to Ultra-Low Attachment Surface 6 well plates (Corning Inc., Corning, NY, USA) in a serum-free DMEM medium containing 10 ng/mL basic fibroblast growth factor (bFGF; Wako, Osaka, Japan), 10 mg/mL human insulin (CSTI, Miyagi, Japan), 100 mg/mL human transferrin (Roche, Basel, Switzerland), and 100 mg/mL bovine serum albumin (BSA; Nacalai Tesque) and incubated at 37 °C in a 5% CO_2_ incubator for 10 days [13]. Tumor spheroids were manually counted under an inverted phase contrast microscope (BZ-X710 Microscope and BZ-X Viewer, BZ-X Analyzer imaging system, Keyence, Osaka, Japan). All morphometric studies were performed by two examiners blinded to treatment conditions.

### Immunoblot analysis

Immunoblot analysis was performed to assess the expression of epithelial-mesenchymal transition (EMT) markers (E-cadherin and vimentin) and previously reported marker genes of CSCs (CD24, CD26, and CD133). The details are given in the Supplemental Data.

### Animal models

Male BALB/c nude mice (5 weeks old) were purchased from CLEA Japan Inc. (Tokyo, Japan) and maintained in a facility under specific pathogen-free conditions. Mice were fed with pathogen-free laboratory chow and allowed free access to autoclaved water in an air-conditioned room under a 12-h light/dark cycle. A total of 2 × 10^6^ cells (MG-parental *n* = 5, MG-OKS *n* = 6, or MG-GFP *n* = 6) in 200 μL of serum-free PBS were subcutaneously injected into the dorsal flank of mice. Consecutively, 8 weeks after cell transplantation, the volume of generated tumors was calculated by the formula 0.5 × (length) × (width)^2^, and all tumors were excised. All morphometric studies were performed by 2 examiners blinded to treatment conditions. The μCT Scanner (R_mCT; Rigaku Mechatronics, Tokyo, Japan) was used for microarchitectural analysis. Parameters used for the scans were as follows: FOV 30 mm, 90 kV tube voltage, and 160 μA tube current.

### Hematoxylin-eosin staining and immunofluorescence staining on frozen sections

Mice were euthanized for histological analyses at 8 weeks after cell transplantation. For analyses, 10-μm-thick frozen sections were cut, washed 5 times with PBS, and stained with hematoxylin-eosin. Sections were microscopically evaluated to confirm the presence of tumor cells. To evaluate the proliferation ability, hypoxia environment, and the osteogenic markers in the xenograft tumor tissues, immunofluorescence staining was performed under a fluorescence microscope (BZ-X710 Microscope and BZ-X Viewer, BZ-X Analyzer imaging system, Keyence) with an anti-human Ki-67 rabbit polyclonal antibody (1:50; Novus Bio, Littleton, CO, USA, catalog number: NB500-170), an anti-human hypoxia-inducible factor 1α (HIF-1α) rabbit polyclonal antibody (1:100; Abcam, Cambridge, MA, USA, catalog number: ab82832), and an anti-human osteocalcin mouse monoclonal antibody (1:100; Takara Bio, Shiga, Japan, catalog number: M184), respectively. The secondary antibodies used were the Alexa-Fluor 594-conjugated chicken anti-rabbit IgG (1:100; Invitrogen, Carlsbad, CA, USA, catalog number: A-21442) for both Ki-67 and HIF-1α and the Alexa-Fluor 594-conjugated goat anti-mouse antibody (1:100; Molecular Probes, Eugene, OR, USA, catalog number: R37121) for osteocalcin, while nuclear staining was performed using the 4′,6-diamino-2-phenylindole (DAPI) solution (1:5000; Sigma-Aldrich). The positivities were quantified using ImageJ software (National Institute of Health, Bethesda, MD, USA) in four randomly selected fields. All morphometric studies were performed by two examiners blinded to treatment conditions.

### Microarray analysis

Total RNA was extracted from the cells 11 days after gene transduction using an RNeasy mini kit (Qiagen). For RNA quality control, concentration and purity were checked with a NanoDrop 1000 (Thermo Fisher Scientific, Waltham, MA, USA) and integrity was evaluated with a TapeStation system (Agilent Technologies, Santa Clara, CA, USA). Gene expression profiling in the MG-parental, the MG-OKS, and the MG-GFP cells was performed using the SuperPrint G3 Human Gene Expression 8 × 60 K Ver3 Microarray (Agilent Technologies) following the manufacturer’s protocol.

The data were analyzed using the GeneSpring 13.1.1 software program (Agilent Technologies) as follows. Threshold raw signals were set to 1.0, and signal intensities were used with log base 2 transformation. The 75th percentile normalization method was used as the normalized algorithm (http://genespringsupport.com/faq/normalization). The default flag setting was used. The number of detected probes in at least one of three cell groups was 36,323, and the probes were used for further analysis. Gene ontology (GO) analysis of the obtained microarray data was also performed using GeneSpring GX 13.1.1 software (Agilent Technologies). *Z*-scores were calculated by subtracting the overall averaged gene intensity from the normalized intensity of each gene and by dividing this result by the standard deviation (SD) of all measured intensities, according to the following equation: *Z*-score = (intensity − mean intensity)/SD [20].

Microarray data have been deposited in NCBI GEO under accession number GSE143556 (https://www.ncbi.nlm.nih.gov/geo/query/acc.cgi?acc=GSE143556).

### Statistical analysis

Obtained results were statistically analyzed using a software package (Graph Pad Prism™, MDF Software, La Jolla, CA, USA). All values were expressed as mean ± SEM (standard error of the mean). Multiple comparisons among groups were made using a one-way analysis of variance (ANOVA) followed by post hoc testing with Tukey’s procedure. A probability value (*P*) of less than 0.05 was considered to denote statistical significance.

## Results

### Transduction of defined factors enhanced CSC properties in OS cells

**Fig. 1:**
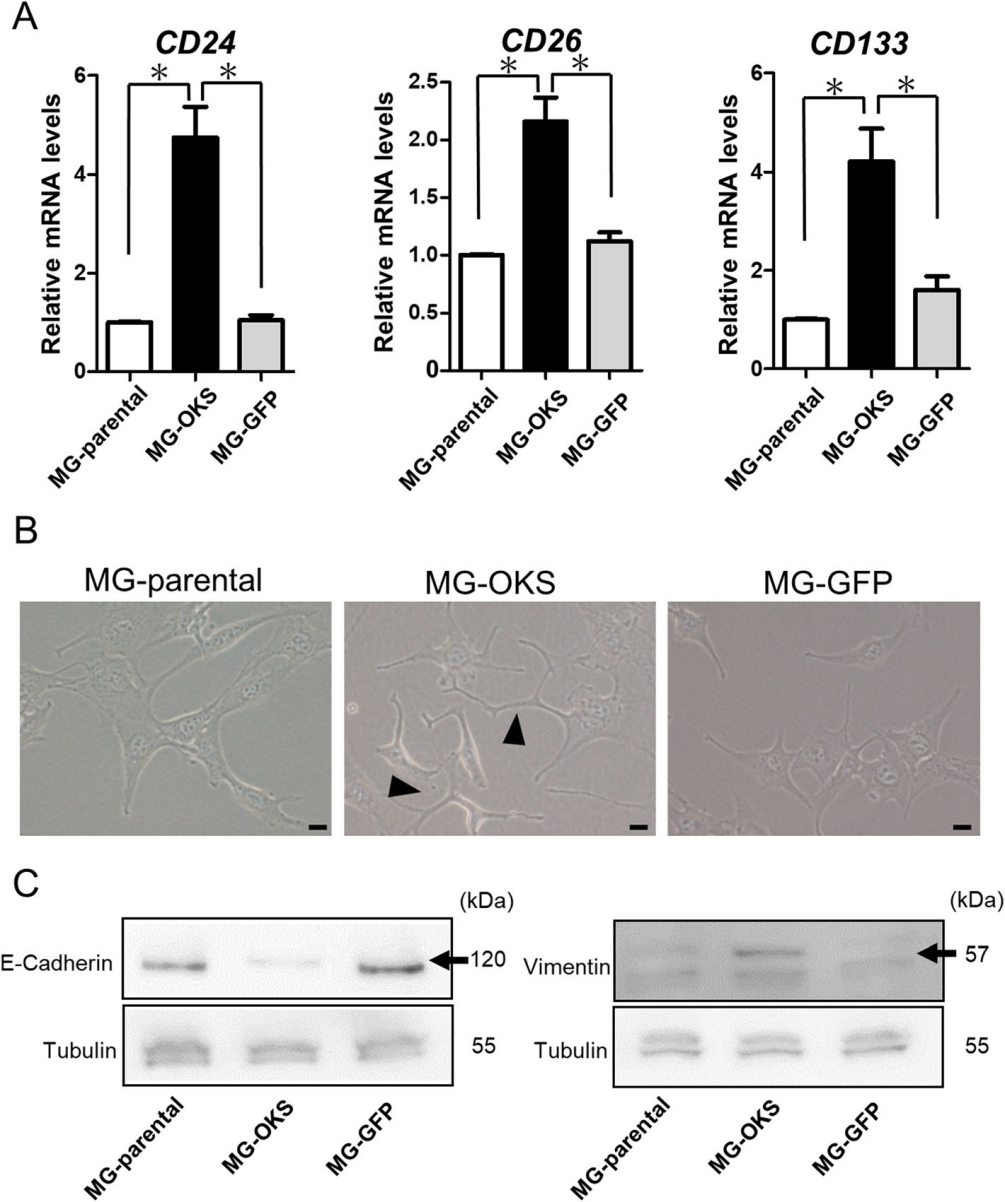
The transduction of OKS induced CSC properties in MG-63 cells in vitro. **a** qRT-PCR of previously reported markers related to CSCs of various cancers in the transduced MG-63 cells. The mRNA expression levels were normalized to those of *β-*actin. The mRNA expression level of MG-parental cells was set to 1. The error bars indicate the standard error of the mean: SEM. *P < 0.05. **b** The morphology of the transduced MG-63 cells was evaluated at 4 days after transduction by phase contrast microscopy. The transduction of OKS led to distinct morphological changes (arrows). Scale bars represent 20 μm. **c** Immunoblot analyses of EMT markers (E-cadherin and vimentin)

The efficiency of retroviral infection was monitored with the expression of GFP in control cells (MG-GFP) using fluorescence microscopy; a strong expression of GFP was accordingly observed (Supplemental Fig. S1B). qPCR analyses revealed that the total transcript levels of *OCT3/4*, *KLF4*, and *SOX2* were significantly elevated in the MG-OKS cells (*P* < 0.05, *n* = 3) (Supplemental Fig. S1C). To assess the stem cell property of the gene-transfected and parental cells, we evaluated the expression levels of previously reported marker genes of CSCs in several cancers, such as *CD24* [21], *CD26*, and *CD133* [22] by qPCR analysis. The mRNA expressions of all the genes were significantly increased in the MG-OKS cells compared with those of other cell populations (*P* < 0.05, *n* = 3) (Fig. 1a). We also evaluated alterations in the markers at the protein level by immunoblot analyses (Supplemental Fig. S1D). Regarding the morphology of the cells, an elongated cell body with invasive processes was observed in the MG-OKS cells, whereas the MG-parental and the MG-GFP cells predominantly consisted of spindle-shaped cells (Fig. 1b). In addition, the EMT, a biological process that assumes stem-like features, was also assessed by immunoblotting. Accordingly, the MG-OKS cells were characterized by a decreased expression of E-cadherin and an increased expression of vimentin (Fig. 1c).

### Decreased proliferation and elevated migration were observed in the MG-OKS cells

**Fig. 2:**
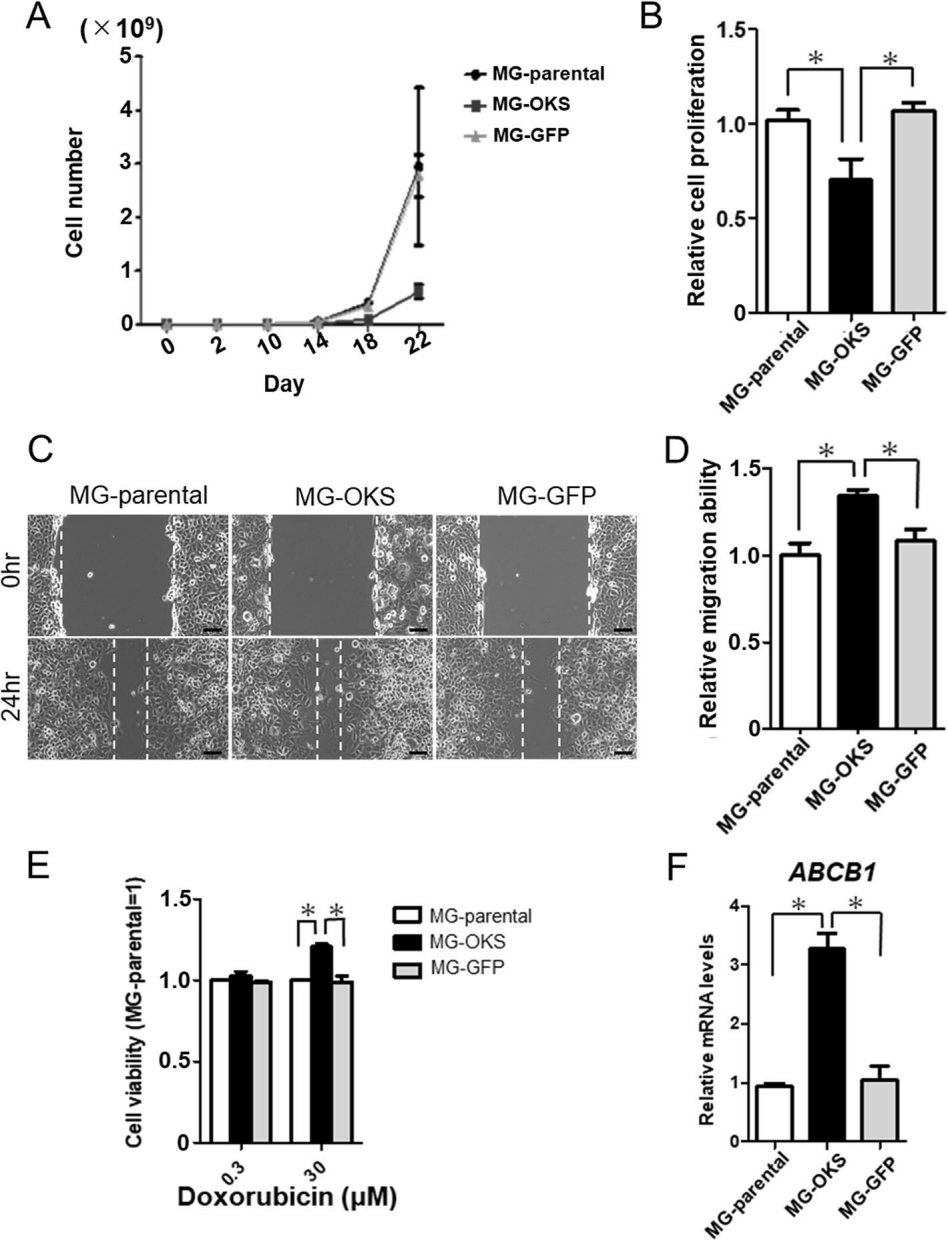
Cell proliferation of the transduced and parental MG-63 cells in vitro. **a** The cell number of transduced MG-63 cells was counted every 4 days from day 10 to day 22 after transduction. **b** Cell proliferation was examined by WST-8 assay. The proliferation rate of MG-parental cells was set to 1. The error bars indicate the standard error of the mean: SEM. *P < 0.05. **c** Representative images of wound healing assays at 0 and 24 h. **d** The effects of transduction on the migration ability of MG-63 cells were determined by a wound healing assay. The migration distance (MD) in each group was calculated according to the following equation: MD = the width of the scratch at 0 h − the width of the scratch at 24 h. The MD value of the MG-parental population was used as a reference. The relative cell migration ability was determined by the following equation: relative cell migration ability = MD (MG-OKS) or MD (MG-GFP)/MD (MG-parental). **e** Doxorubicin-chemoresistance analysis. The viability of cells in the presence of doxorubicin was measured by WST-8 assay. The viability of the MG-parental cells at each concentration was set to 1. **f** mRNA level of *ABCB1* was assessed by qPCR. The mRNA expression levels were normalized to those of *β-*actin, and the mRNA expression level of MG-parental cells was set to 1. The error bars indicate the standard error of the mean: SEM. *P < 0.05

The number of the MG-OKS cells was lower than that of both the MG-parental and the MG-GFP cells at 22 days after gene transduction, but this was not statistically significant (Fig. 2a). Likewise, cell proliferation assays also revealed that the proliferation of cells was significantly decreased in the MG-OKS cells compared with other cells at 72 h after seeding (*P* < 0.05, *n* = 3) (Fig. 2b). In wound healing assays, the speed of wound healing was obviously increased in the MG-OKS group (Fig. 2c) and was significantly elevated in the MG-OKS cells compared with other cells (*P* < 0.05, *n* = 5) (Fig. 2d).

### Transduction of the factors enhanced chemoresistance to DOX in OS cells

To examine the effect of the transduction of *OCT3/4*, *KLF4*, and *SOX2* genes on the chemoresistance to DOX in MG-63 OS cells, we compared the viability of cells after treatment with DOX using the WST-8 assays. There was no significant difference in cell viability following treatment with a low concentration of DOX (0.3 μM) (*P* = 0.3, *n* = 3) (Fig. 2e). However, when a higher concentration of DOX (30 μM) was administered, the viability of MG-OKS cells was approximately 20% higher than that in both the MG-parental and MG-GFP cells (*P* < 0.05, *n* = 4) (Fig. 2e). In addition, the mRNA expression level of ATP-binding cassette subfamily B member 1 (*ABCB1*; also known as *MDR1*) gene, which encodes the membrane drug transporter P-glycoprotein and has been reported as a well-described mechanism of resistance to drugs [23], was approximately 3 times higher in the MG-OKS cells compared with other cells (*ABCB1*: MG-parental set to 1, MG-OKS 3.4 ± 0.3, MG-GFP 1.1 ± 0.3, *P* < 0.05; *n* = 3) (Fig. 2f).

### Confirmation of the reproducibility with another osteosarcoma cell line

We confirmed the reproducibility of our results using another osteosarcoma cell line (NOS-1). Several in vitro experiments, including measurement of the mRNA expression of markers (*CD24*, *CD26*, and *CD133*), cell morphology, cell proliferation rate, migration ability, and chemoresistance to DOX, were performed. We obtained similar results as in MG-63 cells. The mRNA expression of *CD24* was significantly increased in the NOS-OKS cells compared to that in other cell populations (*P* < 0.05, *n* = 3). The mRNA expression of *CD26* and *CD133* were significantly increased in NOS-OKS cells compared with NOS-parental cells (*P* < 0.05, *n* = 4) (Fig. S2A). For morphological features, NOS-OKS cells showed similar changes as in MG-OKS cells (Fig. S2B). Cell proliferation assays revealed significantly decreased proliferation in NOS-OKS cells (*P* < 0.05, *n* = 4) (Fig. S3A); in wound healing assays, the rate of wound healing was significantly increased in NOS-OKS cells compared with other cells (*P* < 0.05, *n* = 5) (Fig. S3B, C). Chemoresistance to DOX and the mRNA expression level of *ABCB1* was significantly higher than those in both NOS-parental and NOS-GFP cells, which were similar to those in the experiments using MG-63 (*P* < 0.05, *n* = 3) (Fig. S3D, E).

### Osteogenic ability was elevated in the MG-OKS cells

**Fig. 3:**
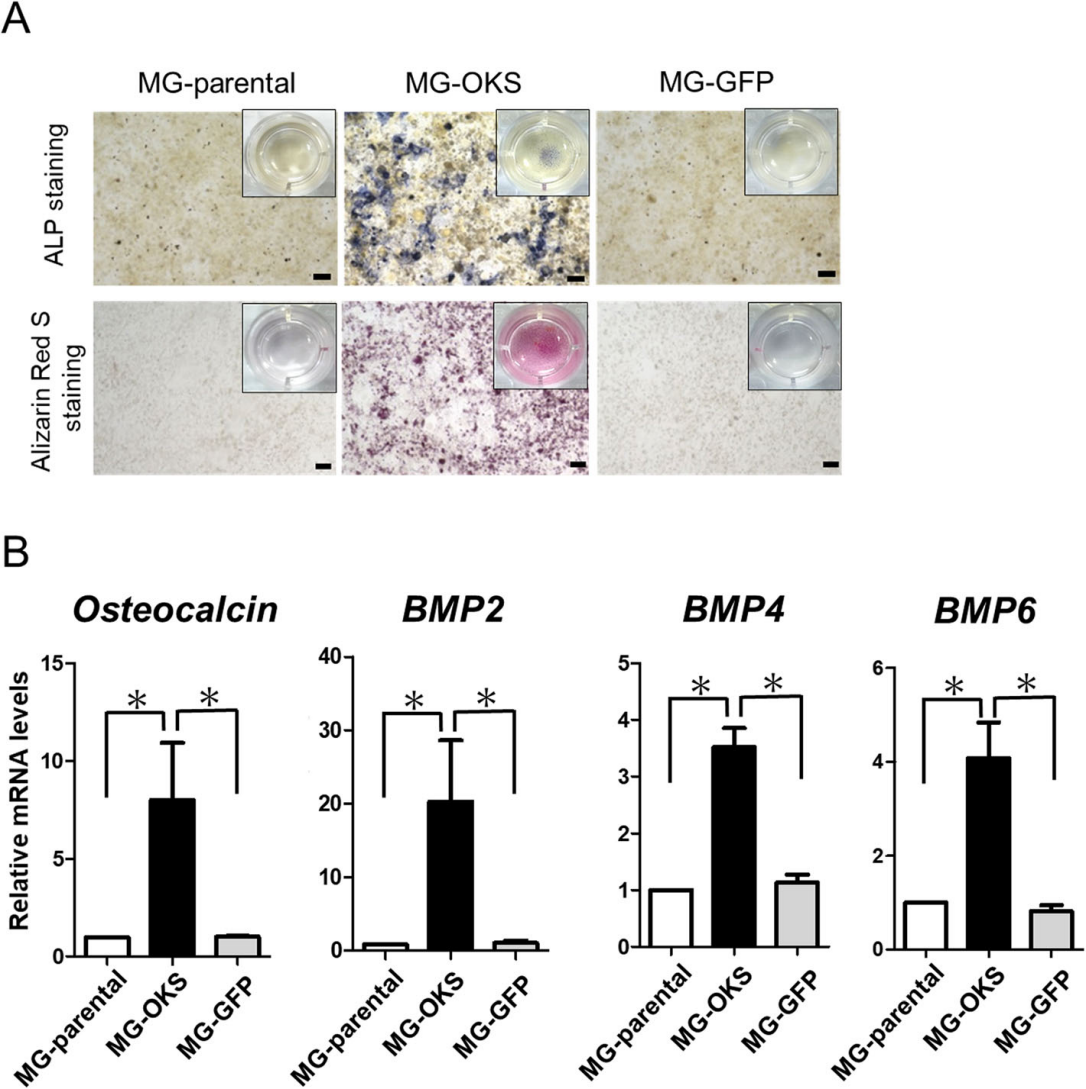
Osteogenic differentiation of MG-63 cells cultured in osteogenic induction medium. **a** Representative images of alkaline phosphatase (ALP) staining and Alizarin Red S staining (inset: macroscopic images). **b** qRT-PCR of markers related to bone formation (*osteocalcin* and *BMP-2*, *BMP-4*, and *BMP-6*). The mRNA expression levels were normalized to those of *β-*actin, and the mRNA expression level of MG-parental cells was set to 1. The error bars indicate the standard error of the mean: SEM. *P < 0.05

Osteosarcoma has been generally defined as a high-grade tumor of malignant mesenchymal cells with osteoid formation [17]. Hence, we assessed the ability of osteogenic differentiation in the cells. After culturing with the osteogenesis culture kit for 3 weeks, strong staining of both ALP and Alizarin Red S was observed in the MG-OKS cells but not in the other cells (Fig. 3a). Concomitantly, the mRNA expressions of both *osteocalcin*, a marker of mature osteoblasts [24], and *BMPs* (*BMP-2*, *BMP-4*, and *BMP-6*), which are known to stimulate osteoblast differentiation [18, 19], were significantly elevated in the MG-OKS cells compared with other cells (*osteocalcin*: MG-parental set to 1, MG-OKS 6.7 ± 3.0, MG-GFP 1.0 ± 0.05, *P* < 0.05, *n* = 5; *BMP2*: MG-parental set to 1, MG-OKS 23.3 ± 9.9, MG-GFP 1.1 ± 0.2, *P* < 0.05, *n* = 5; *BMP4*: MG-parental set to 1, MG-OKS 3.5 ± 0.3, MG-GFP 1.1 ± 0.1, *P* < 0.05, *n* = 3; *BMP6*: MG-parental set to 1, MG-OKS 4.0 ± 0.6, MG-GFP 0.8 ± 0.1, *P* < 0.05, *n* = 3) (Fig. 3b).

### The sphere formation ability was elevated in the MG-OKS cells

**Fig. 4:**
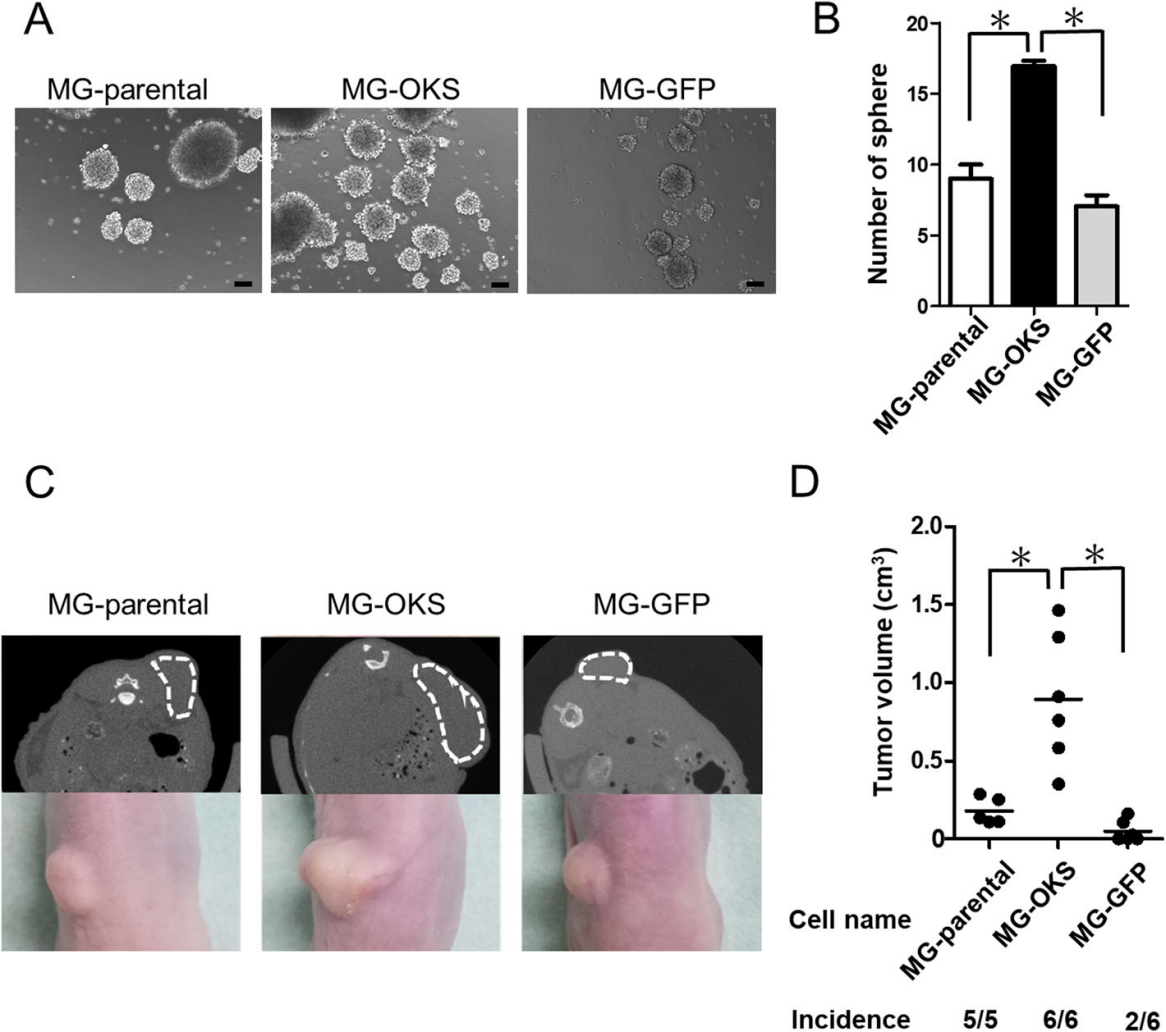
The sphere formation ability in vitro and tumorigenicity in vivo. **a** Representative images of sphere formation assay after culturing on low attachment dishes with serum-free medium for 10 days. **b** The number of spheres was counted under microscopy. The error bars indicate the standard error of the mean: SEM. Scale bars represent 100 μm. **c** Representative μCT images of mice 8 weeks after implantation and tumors derived from each cell group (dotted white line in upper panel). Photographs of representative mice and tumors (lower panel). **d** The tumorigenicity of the cells after implantation in the subcutaneous regions of immunodeficient nude mice. A total of 2 × 10^6^ cells were subcutaneously injected into flank of immunodeficient nude mice on day 13. The volume of the tumors was calculated by the formula 0.5 × (length) × (width)^2^. The red bars indicate the median tumor volume. The error bars indicate the standard error of the mean: SEM. *P < 0.05

As previously reported, it is known that CSC has the ability to form sphere in anchorage-independent and serum-starved conditions [25, 26]. To examine the sphere formation ability of these cells, we performed sphere formation assay (Fig. 4a). In the MG-OKS cultures, the number of spheres was significantly increased compared to that of other cells (Fig. 4b).

### In vivo OS tumorigenicity was accelerated in the MG-OKS cells

**Fig. 5:**
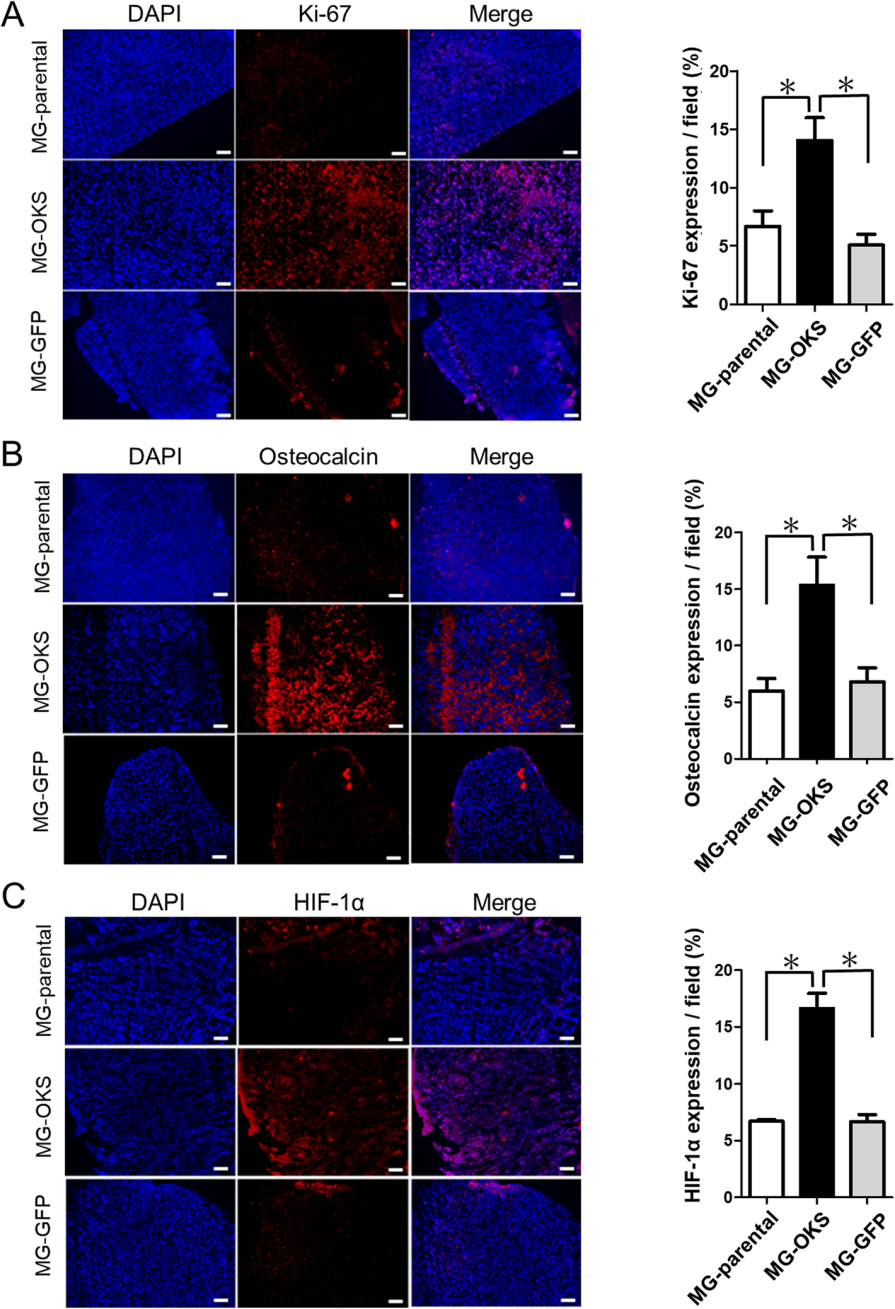
The histology of the xenografts derived from the transduced and parental MG-63 cells. **a**–**c** Representative images of immunofluorescence analysis (left panel). The tumors derived from MG-OKS were positive for Ki-67, osteocalcin, and HIF-1α (red). Nuclei were stained with blue fluorescent DAPI. Positive areas were assessed by software, 4 fields were randomly selected, and means were calculated (right panel). Scale bars 100 μm. The error bars indicate the standard error of the mean: SEM. *P < 0.05

To examine their in vivo tumorigenicity, we subcutaneously transplanted parental and gene-transduced OS cells into the dorsal area of immunodeficient nude mice. In mice with MG-OKS xenografts, 100% engraftment and a significantly larger volume of tumors were observed at 8 weeks after cell transplantation compared with those carrying xenografts derived from MG-parental and MG-GFP (*P* < 0.05) (MG-parental, 0.1 ± 0.08 cm^3^, MG-OKS 0.8 ± 0.1 cm^3^, MG-GFP 0.04 ± 0.02 cm^3^, *P* < 0.001; *n* = 5 in MG-parental, *n* = 6 in MG-OKS, and MG-GFP) (Fig. 4c, d). Immunofluorescence staining revealed a strong staining of Ki-67, a marker of proliferative potential, in tumors derived from MG-OKS cells (Fig. 5a). We also observed increased positive staining of both osteocalcin (Fig. 5b) and HIF-1α, which permits hypoxic tumor cells to upregulate proteins that promote their survival and increase their aggressiveness [27], in tumors derived from MG-OKS cells (Fig. 5c).

### Microarray and data analyses

**Fig. 6:**
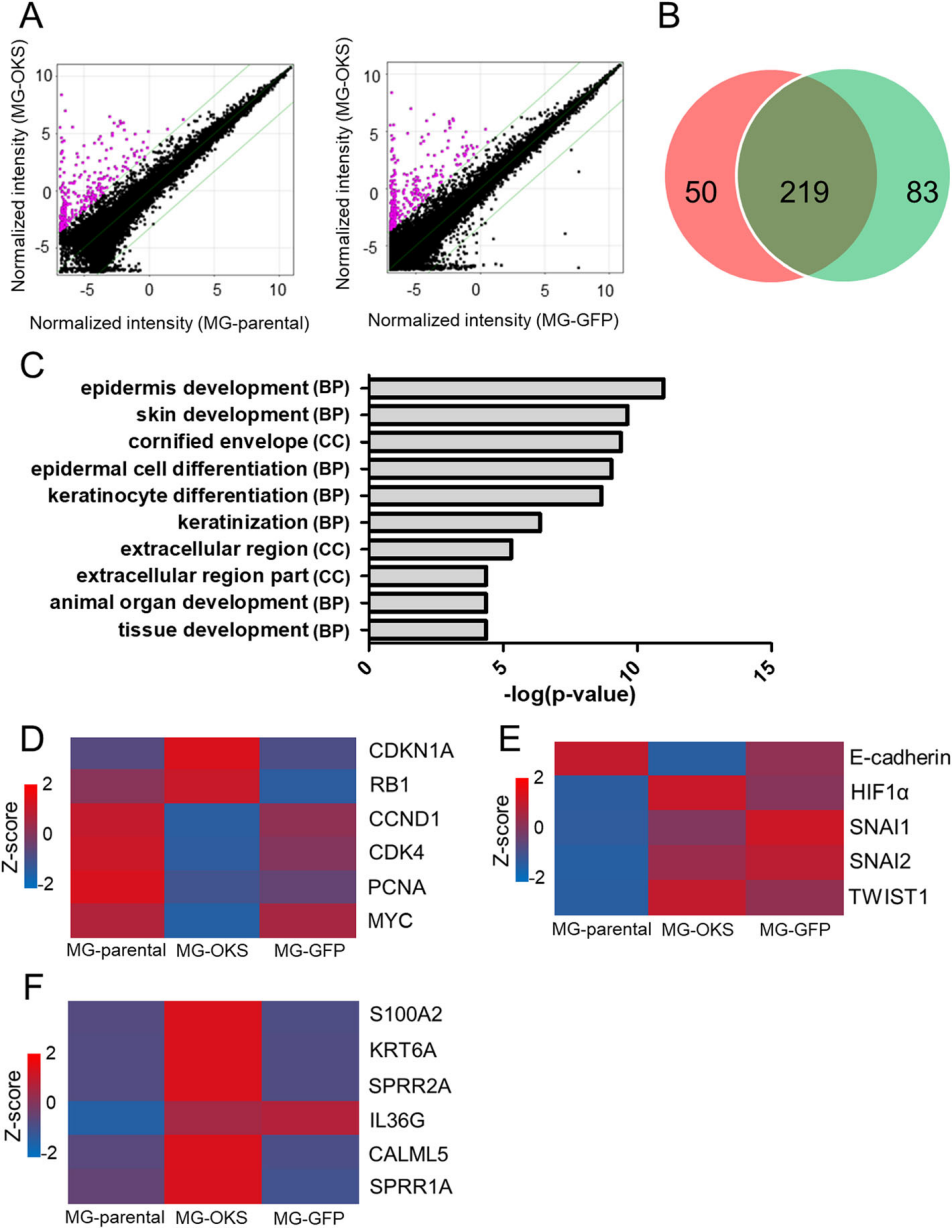
Gene expression microarray analysis of the transduced and parental MG-63 cells. **a** Scatter plot showing the 10-fold upregulated genes (the pink dots). **b** Venn diagram showing the number of the 10-fold upregulated genes in the comparison between MG-OKS vs MG-parental (left), MG-OKS vs MG-GFP (right), and the number of genes that are differential expression in both comparisons (center). **c** GO analysis result of upregulated 219 genes in MG-OKS cells. The enriched GO terms are ranked according to a *P* value. Categories of each GO term (BP: biological process, CC: cellular component) were shown in parentheses. **d** Heatmap showing the normalized gene signal intensity related to cell proliferation transformed into *Z*-score. **e** Heatmap showing the normalized gene signal intensity related to EMT and migration transformed into *Z*-score. **f** Heatmap showing the normalized gene signal intensity related to epithelial differentiation transformed into *Z*-score

We used microarray analysis to identify changes in gene expression pattern among the MG-parental, MG-OKS, and MG-GFP cells. The effects were visualized after filtering by applying the gene list to a scatter plot with a fold-change level of 10 as a threshold (genes upregulated, > 10-fold change) (Fig. 6a). In the MG-OKS cells, 269 and 302 genes were upregulated above a 10-fold change compared with the MG-parental and the MG-GFP cells, respectively. Among these upregulated genes, 219 genes were commonly upregulated as compared with both the MG-parental and the MG-GFP cells (Fig. 6b). Of these 219 genes, the top 10 differentially expressed genes are shown in Table 1. In contrast, 75 and 86 genes showed over 10-fold downregulation in the MG-OKS cells compared with MG-parental and MG-GFP cells, respectively (Fig. S4A). Among these downregulated genes, only 1 gene was commonly downregulated (Fig. S4B). The gene was *C3orf49*, which function had been still unclear; therefore, we focused on the upregulated 219 genes. GO analysis showed that the 219 upregulated genes were mainly enriched in “biological processes (BP),” including epidermis development (GO: 0008544), skin development (GO: 0043588), epidermal cell differentiation (GO: 0009913), keratinocyte differentiation (GO: 0030216), keratinization (GO: 0031424), animal organ development (GO: 0048513), tissue development (GO: 0009888), and followed in “cellular component (CC),” including cornified envelope (GO: 0001533), extracellular region (GO: 0005576), and extracellular region part (GO: 0044421) (Fig. 6c).

**Table 1:** The top 10 differentially expressed genes, which were upregulated in the MG-OKS cells

Gene name	Fold change	
	MG-parental vs MG-OKS	MG-OKS vs MG-GFP
KRT6A	15.2	− 15.3
SPRR2A	12.4	− 12.6
IGF2	13.5	− 12.4
KRT6C	11.5	− 12.3
NCF2	11.4	− 11.5
CALB1	11.0	− 11.1
LINC02582	11.0	− 11.1
GJB4	10.2	− 10.3
LINC00520	10.1	− 10.2
KRT6B	10.1	− 10.2

MG-parental vs MG-OKS: Log_2_ (signal intensity of MG-OKS/signal intensity of MG-parental)

MG-OKS vs MG-GFP: Log_2_ (signal intensity of MG-GFP/signal intensity of MG-OKS)

*Abbreviation*s: *KRT6A* keratin 6A, *SPRR2A* small proline-rich protein 2A, *IGF2* insulin-like growth factor 2, *KRT6C* keratin 6C, *NCF2* neutrophil cytosol factor 2, *CALB1* Calbindin 1, *GJB4* gap junction beta-4 protein, *KRT6B* keratin 6B

## Discussion

In our series of experiments, we have succeeded in obtaining for the first time CSC-like cells from a human OS cell line by transducing them with the *OCT3/4*, *KLF4*, and *SOX2* genes. Recently, CSCs, a subpopulation of tumor cells, have been characterized and emerged as a major topic of interest in the cancer research field [28]. Isolation and subsequent studies of CSCs from various types of cancers indicated CSCs as one of the crucial causes of conventional treatment failure. In consequence, targeting CSCs should be a promising perspective for the development of more effective anticancer therapies. Especially, CSCs are attracting a great deal of attention in the research subjects of rare cancers, such as OS. CSCs are considered to play a key role for the poor prognosis of patients with OS due to therapeutic resistance, and the possible reason for the development of recurrence or metastasis [4]. However, difficulties in acquiring proper research samples by conventional methods have interfered with the elucidation of OS CSC biology and the development of novel therapies that could target OS CSCs. Several previous studies have attempted to isolate OS CSCs from clinical specimens [13–17]. First, CSCs were collected by using their self-renewal capacity to form spherical colonies, named “sarcospheres,” under serum-free conditions [25, 26]. The formation of sarcospheres was shown to be further improved by cultivating under the hypoxic conditions of the tumor microenvironment [29]. Then, OS CSCs were isolated by sorting cells according to the expression of previous reported markers associated with cancer stem cells, such as CD133 or CD117, in combination with Stro-1 [30]. Other methods used to isolate OS CSCs included the identification of a “side population” of cells able to efflux Hoechst dyes [31], with/without the expression of surface markers, such as CD117 or STRO-1 [32]; the sorting of cells with high ALDH1 activity; and the tracking of subpopulations of *OCT-4-*positive cells [33]. In this study, we successfully generated OS CSC-like cells by transducing defined factors into human OS cell lines. Unlike the previous methods, our novel technique could make it feasible to obtain OS CSCs more effectively and abundantly.

CSCs are predominantly present in the dormant state of the cell cycle and undergo proliferation in response to physiological cell stimuli; the quiescent state is an important factor in chemoresistance [34]. Our data revealed a slow cell proliferation rate in vitro and high tumorigenicity in vivo (larger tumor volume with more Ki67+ tumor cells) in MG-OKS xenografts. Subcutaneous transplantation of the cells may have led to cell cycle re-entry from the dormant state as external stimuli. Dembinski et al. reported that a subpopulation of slow cycling cells in the pancreas adenocarcinoma cell lines exhibited increased tumorigenic and invasive potentials in vivo [35]; our results are consistent with their findings. We assessed altered expression of genes, related to slow cell growth in MG-OKS cells using microarray data. Our data revealed increased expression of the cyclin-dependent kinase (CDK) inhibitor 1A (CDKN1A) and retinoblastoma 1 (RB1) and decreased expressions of CDK4, cyclin D (CCND), proliferating cell nuclear antigen (PCNA), and MYC (Fig. 6d). CDKN1A inhibits the cell cycle during G1 phase [36]. CCND binds to and activates CDK4, and then the CCND-CDK4 complex inhibits members of the RB protein family including RB1 and regulates the cell cycle during G1/S transition [37]. Therefore, slow cell growth in MG-OKS cells was likely caused by inhibition of the cell cycle at the G1 phase via increased CDKN1A expression. Decreased expression of PCNA which accumulate in the G1 phase of the cell cycle, reaching maximum expression in S phase, may also inhibit the G1/S transition [38]. MYC is one of the most commonly activated oncogenes implicated in the pathogenesis of human cancers and induces tumorigenesis by evading multiple tumor-suppressing checkpoint mechanisms including proliferative arrest [39]. Our results showed decreased MYC expression, which likely contribute to the slow cell proliferation of MG-OKS cells in vitro.

As previously reported, isolated OS CSCs have been associated with the metastasis and chemoresistance of the disease [29, 32, 40]. And, this increased chemoresistance of CSCs has been associated with their increased ability for DNA repair, their frequent quiescent state of low proliferation rate [41], suppression of the apoptotic signaling, and increased levels of lysosomal activity due to the overexpression of vacuolar ATPases [42]. Additionally, chemoresistance of CSCs might be strongly related to a gain in the drug efflux capability due to the overexpression of the ATP-binding cassette (ABC) family transporters, especially *ABCG2* [43] or the *ABCB1* multidrug resistance P-glycoprotein [44–46]. In the current study, our generated OS CSC-like cells showed enhanced chemoresistance to doxorubicin, the most important anticancer drug in the chemotherapy of OS [47], associated with an increased mRNA expression of *ABCB1*. Therefore, it is suggested that OS CSCs have specific characteristics making them more resistant to chemotherapies. The present results also indicated that the generated OS CSC-like cells showed elevated ability for cell migration, which is associated with both the progression and metastasis of OS cells.

Osteosarcoma is a neoplasm derived from the primitive bone-forming mesenchyme. Pathologic feature is characterized by the production of osteoid and new bone by spindle-shaped tumor cells. However, the MG-63 cell line, a human OS-derived cell line, has impaired the capability to synthesize a correct extracellular bone matrix [48, 49] and has a low ALP activity [50]. Interestingly, in the current study, the CSC-like cells generated from MG-63 cells following transduction of defined factors were able to induce osteogenic differentiation in vitro, whereas bone formation was not observed in vivo. There was a report that bone formation was not observed following simple implantation of an OS cell line into muscle tissue of mice; however, formation of the bone was noted when cells were pretreated with dexamethasone before implantation [51]. One of the possible explanations for our in vivo results should be that bone formation did not occur because steroids derived from nude mice could not act on human-derived OS cells. Another report suggested that the γ-carboxylation of osteocalcin by γ-carboxylase increased its affinity for hydroxyapatite, the mineral component of the bone extracellular matrix [52]. The absence of bone formation despite the increased expression of osteocalcin both in vitro and in vivo suggested a problem with this carboxylation process.

The EMT is a biological process including various phases through which epithelial cells lose their characteristics with intercellular adhesion and proliferative capacity while acquiring mesenchymal characteristics with increased migratory and invasive potencies. This process has been reported in several cancers of epithelial origin and is important to metastatic tumor cell progression and chemoresistance [53]. In contrast, the precise role of EMT-related processes of tumor mesenchymal origin, such as OS, is still mostly unknown [53]. There are reports that activation of the EMT and reactivation from cellular dormancy could cause tumor metastasis and/or relapse [54, 55]. In this study, we reported that the MG-OKS cells showed a decreased expression of E-cadherin with an increased expression of vimentin, suggesting activation of EMT. Yang et al. reported that hypoxia or HIF-1α overexpression induces various EMT regulators, such as SNAI1, SNAI2, and TWIST, and that TWIST plays critical roles in the acquisition of EMT and metastatic phenotypes including an increased migration ability [56]. Our microarray analysis results are consistent with this previous report, as MG-OKS cells showed greater migration ability with increased expression of HIF-1α and TWIST and decreased expression of E-cadherin (Fig. 6e).

We also surveyed the differentially expressed genes between MG-OKS and other cells by microarray analysis, and numerous genes showing significant differential expression were identified. Interestingly, although OS is a tumor of mesenchymal origin, upregulated genes in MG-OKS cells included epithelial-related genes, such as small proline-rich protein 2A (*SPRR2A*), *SPRR1A*, keratin 6A (*KRT6A*), and keratin 6B (*KRT6B*)*.* There have been several reports on the relationships of keratin and SPRRs with cancer [57]. Zachary et al. reported that urothelial cell carcinomas expressed high levels of the basal keratins (*KRT6A*, *KRT6B*, *KRT6C*, *KRT14*, and *KRT16*), which are not normally expressed in the urothelium, and expression of these genes was an indicator of poor prognosis [57]. Kim et al. reported that *SPRR3* promoted the proliferation of breast cancer cells by enhancing p53 degradation via the AKT and MAPK pathways [58]. Vos et al. reported that a subset of genes, such as *SPRR2A*, *KRT6A*, *S100A2*, and others, which are related and form an effective barrier against external (chemical and physical) stimuli, were expressed in bronchial epithelial cells and keratinocytes [59]. Our microarray data revealed similar changes in these genes (Fig. 6f). However, few studies have reported on the relationship between stromal cell-derived OS and epithelial markers, and how these associated genes forming a barrier in the epithelial system affect OS CSC-like cells remains unclear. So further study of these relationships might lead to the discovery of prognostic markers and new therapeutic strategies to target OS CSCs.

There have been several limitations in this study. First, to avoid heterogeneity, we designed a polycistronic retroviral vector in which the frames of *OCT3/4*, *KLF4*, and *SOX2* were linked via T2A sequences. Although these genes were individually reported to be correlated with malignant behavior and poor prognosis in OS [33, 60, 61], it has been unclear which gene contributes the most toward gaining of CSC properties. To solve this problem, it will be necessary to use 3 viral vectors independently carrying *OCT3/4*, *KLF4*, or *SOX2* for the generation of CSCs in future studies. Second, it is important to mention that generated OS CSC-like cells might be heterogeneous, with different subpopulations carrying different genetic alterations existing within a tumor. Moreover, these subpopulations might be highly dynamic and able of processes of dedifferentiation and phenotype switching which may render the CSCs resistant to a specific CSC therapy [62]. In this regard, to overcome this limitation, we need to find appropriate culture conditions to maintain CSC properties and future therapies should combine different treatments to target both non-CSCs and CSCs. Finally, similar to normal stem cells, specific signaling or tumor niches might have responsibility for the regulation of OS CSCs [63]. OS CSC niche is reported to be composed of three types of niches within the bone microenvironment where these signaling pathways are particularly active: the perivascular niche, the hypoxic niche, and the endosteal niche [64]. In addition, Wnt and Notch signaling pathways have also been reported to play a role in the progression of OS [65]. In OS cell lines, inhibition of the Wnt and Notch pathways enhanced the sensitization to chemotherapy [66]. Besides this, activation of the Wnt/β-catenin signaling has been reported in the OS CSC population and has been related to the overexpression of *SOX2* and cancerogenesis [67]. Therefore, there is a need for developing and testing therapeutic strategies directed against specific signaling or tumor niches, or both. Although it is true that we need to examine CSC-specific treatments that would target multiple pathways altered in different subsets of CSCs within the tumor in future studies, our novel method could be useful in shedding light on the undisclosed molecular mechanisms and facilitating development of new therapeutic approaches for the management and treatment of human OS.

## Conclusion

To the best of our knowledge, this is the first study to generate CSC-like cells in terms of the elevated expression of CSC markers, slow cell proliferation, higher chemoresistance, increased migration, and osteogenic abilities in vitro, as well as tumorigenicity in vivo, from an established OS cell line by exogenous expression of *OCT3/4*, *KLF4*, and *SOX2* genes. Although further investigations are needed to elucidate the properties of these CSC-like cells, this method could be useful in solving the quantitative problem of CSCs and in elucidating the molecular mechanisms and identifying novel effective therapeutic targets in human OS.

## Supplementary information

**Additional file 1.** Supplementary Data

**Additional file 2.** Supplementary Data. Materials and Methods

**Additional file 3: Table S1.** Primer sequences used in qRT-PCR.

## Data Availability

Microarray data have been deposited in NCBI GEO under accession number GSE143556 (https://www.ncbi.nlm.nih.gov/geo/query/acc.cgi?acc=GSE143556).

## Abbreviations

ABCB1: ATP-binding cassette subfamily B member 1
ALP: Alkaline phosphatase
BMP: Bone morphogenetic protein
bFGF: Basic fibroblast growth factor
BSA: Bovine serum albumin
CALB1: Calbindin 1
CCK-8: Cell Counting Kit-8
CCND: Cyclin D
CDK: Cyclin-dependent kinase
CDKN1A: Cyclin-dependent kinase 1A
CSC: Cancer stem cell
DAPI: 4′,6-Diamino-2-phenylindole
DOX: Doxorubicin
EMT: Epithelial-mesenchymal transition
GJB4: Gap junction beta-4 protein
GO: Gene ontology
GSK3B: Glycogen synthase kinase 3 beta
HIF-1α: Hypoxia-inducible factor 1α
IGF2: Insulin-like growth factor 2
IL-6: Interleukin-6
KLF4: Kruppel-like factor 4
KRT: Keratin
NCF2: Neutrophil cytosol factor 2
OCT3/4: Octamer-binding transcription factor 3/4
OS: Osteosarcoma
PCNA: Proliferating cell nuclear antigen
RT-qPCR: Real-time quantitative reverse-transcription polymerase chain reaction
SOX2: SRY-box transcription factor 2
SPRR: Small proline-rich protein
