# Supplementary figures and images for: Acquisition of cancer stem cell properties in osteosarcoma cells by defined factors
Source: Stem Cell Res Ther. 2020 Oct 2;11:429. doi: 10.1186/s13287-020-01944-9 (PMC7532109; doi:10.1186/s13287-020-01944-9)

Supplementary Data

Figure S1

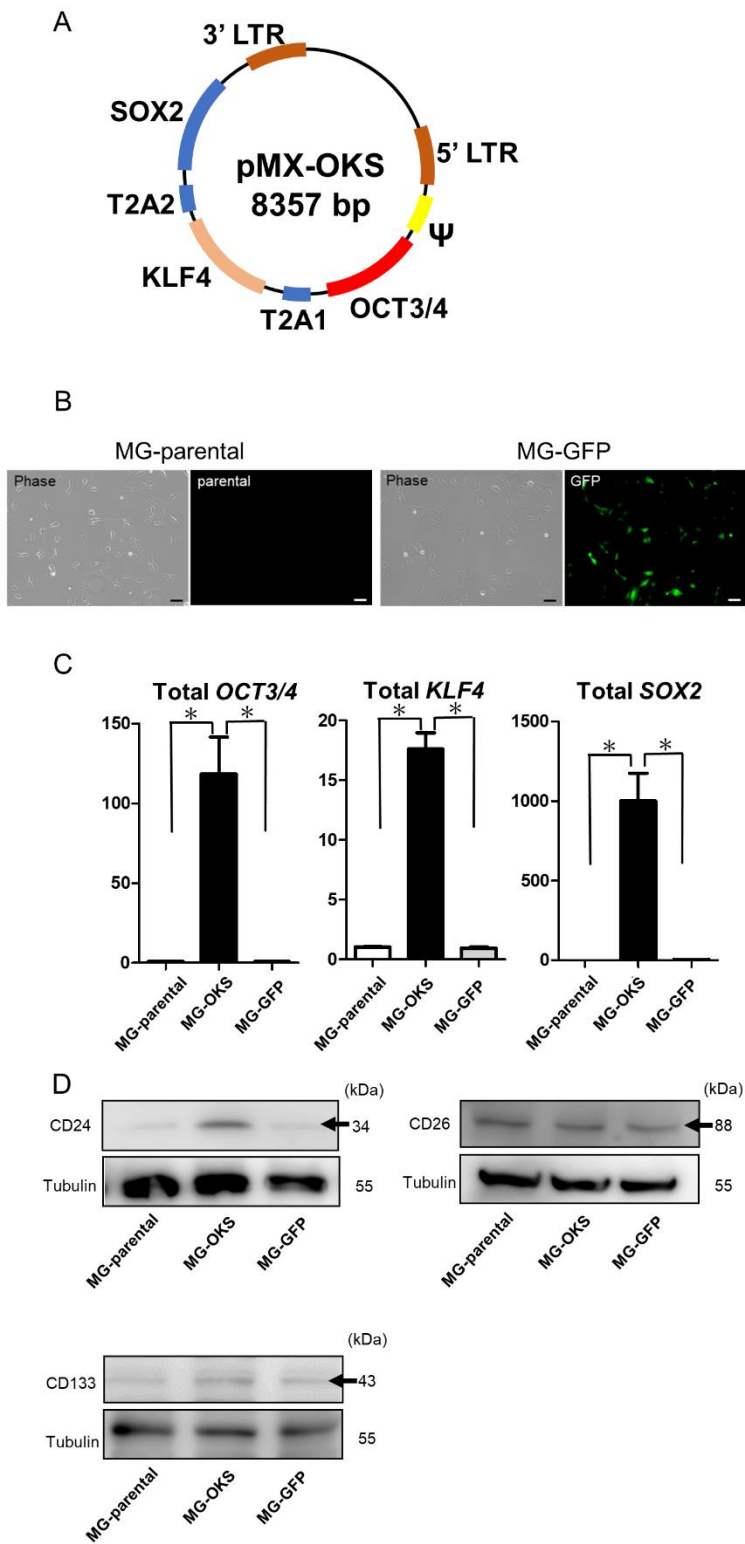

Figure S2

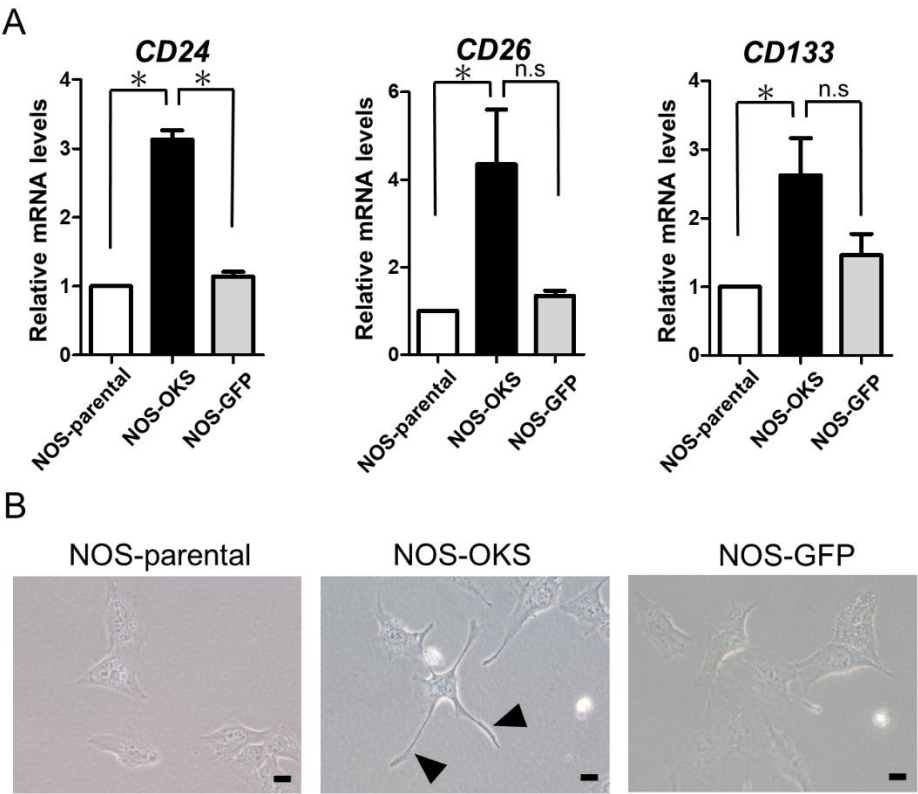

Figure S3

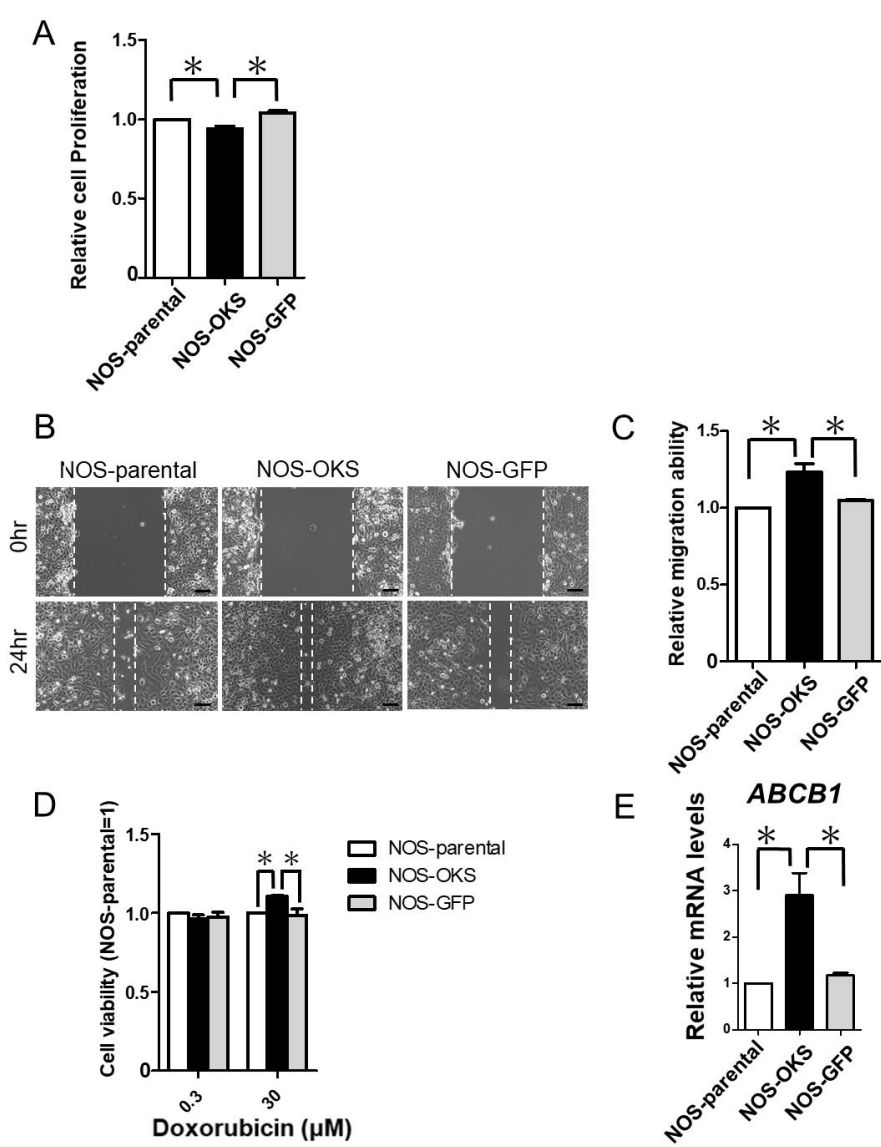

Figure S4

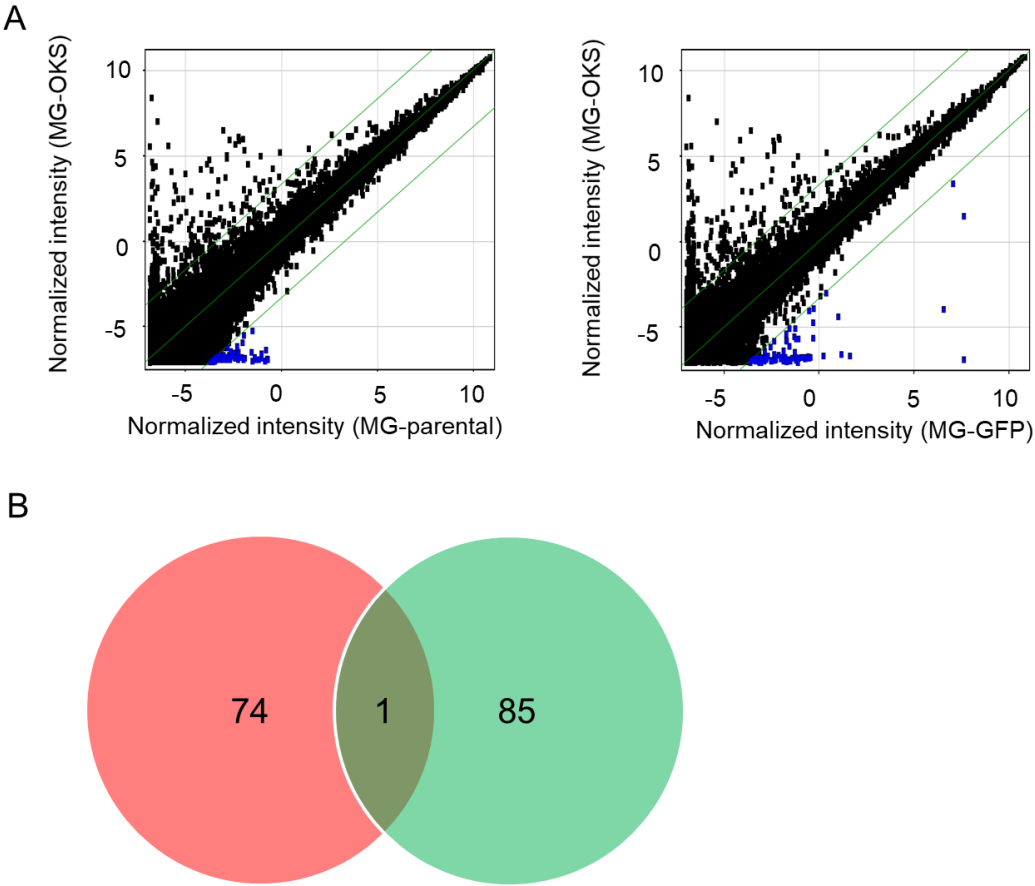

Supplement: Supplementary file 1 — Additional file 1. Supplementary Data [file 13287_2020_1944_MOESM1_ESM.pdf]
