# Supplementary material for: Acquisition of cancer stem cell properties in osteosarcoma cells by defined factors
Source: Stem Cell Res Ther. 2020 Oct 2;11:429. doi: 10.1186/s13287-020-01944-9 (PMC7532109; doi:10.1186/s13287-020-01944-9)
Supplement: Supplementary file 2 — Additional file 2. Supplementary Data. Materials and Methods [file 13287_2020_1944_MOESM2_ESM.docx]

**Supplementary Data**

**Materials and Methods**

***RNA isolation and real-time quantitative reverse-transcription polymerase chain reaction (RT-qPCR)***

Oligo (dT)-primed first-stand cDNA was synthesized using a high-capacity cDNA transcription kit (Applied Biosystems, Foster City, CA, USA). qPCR reactions were performed in 20 μL reaction mixture volumes using the SYBR Green master mix reagent (Applied Biosystems) on the ABI prism 7500 sequence-detection system (Applied Biosystems). PCR conditions were as follows: one cycle at 95 °C for 10 minutes, followed by 40 cycles at 95 °C for 15 seconds, and 60 °C for 1 minutes. Obtained values were normalized to *β-actin*, and the relative expression was analyzed using the ΔΔCt method [1]. Relative mRNA expressions of transduced genes (*OCT3/4*, *KLF4* and *SOX2*), previously reported markers (*CD24*, *CD26* and *CD133*), chemoresistance related gene (*ABCB1*), osteogenic differentiation related genes (*Osteocalcin*, *BMP2*, *BMP4*, and *BMP6*) were assessed. Primers were designed according to the sequences obtained from the GenBank database (Table S1).

***Cell proliferation assay***

Protocol of cell proliferation assay is an established method of detecting and tracking live cells, as previously described [2]. The number of cells was counted every 4 days starting from the 10 days after retroviral transduction. For the WST-8 assay, cells at 10 days after retroviral transduction were seeded in 96-well culture plates at a density of 5×10^3^ cells/well in 100 μL culture media. After incubation for 72 hours, 10 μL of the CCK-8 solution was added, and the optical density was measured at 450 nm using a Model 680 Microplate Reader (Bio-Rad, Hercules, CA, USA). Accordingly, the relative number of viable cells in each well was calculated.

***Cell migration assay***

Cells were seeded in 6-well culture plates at a density of 2×10^5^ cells/well in 2 mL culture medium, and allowed to reach 80 % confluence. A wound was artificially created by scratching the cell monolayer with a 200 μL pipette tip. Plates were washed with phosphate buffered saline (PBS, Takara Bio) to remove detached cells, and were maintained in DMEM with 2% FBS (serum-reduced culture media) for 24 hours [3]. Wound closure was observed at 0 and 24 hours, and images were captured under a microscopy system (BZ-X710 Microscope and BZ-X Viewer, BZ-X Analyzer imaging system, Keyence). The migration distance (MD) in each group was calculated according to the following equation: MD = the width of the scratch at 0 hours - the width of the scratch at 24 hours. The MD value of the MG-parental population was used as a reference. The relative cell migration ability was determined by the following equation: Relative cell migration ability = MD (MG-OKS) or MD (MG-GFP)/MD (MG-parental) [4].

***Immunoblot analysis***

Cells were lysed using the M-PER Mammalian Protein Extraction Reagent (Thermo Fisher Scientific, Rockford, IL, USA). Soluble proteins were collected after centrifugation at 20000 g for 15 minutes at 4 °C. Equal amounts of protein (15 μg) were mixed with electrophoresis sample buffer and boiled for 5 minutes before loading onto a 7.5–15.0 % polyacrylamide gel. Cell lysates were subjected to SDS-polyacrylamide gel electrophoresis (SDS-PAGE) and transferred onto polyvinylidene difluoride (PVDF) membranes (Millipore, Billerica, MA, USA). Membranes were blocked with 5 % nonfat dry milk (Bio-Rad) in Tris-buffered saline Tween- 20 (TBST), and then incubated overnight at 4 °C with primary antibodies in the CanGet Signal Solution 1 (TOYOBO Co., LTD, Osaka, Japan). Following washes, membranes were probed with horseradish-peroxidase (HRP)-conjugated secondary antibodies in the CanGet Signal Solution 2 (TOYOBO Co.) at 25 °C. Proteins were visualized with SuperSignal West Femto, using enhanced chemiluminescent substrate (Thermo Scientific), and the chemical luminescence reaction was detected by the Chemilumino analyzer Las-3000 mini (Fuji film, Tokyo, Japan). Membranes were reprobed with a mouse anti-human α-tubulin antibody (1:1000; Sigma-Aldrich) to confirm equal protein loading. The following primary antibodies were used: a mouse anti-human Vimentin (1:1000; GeneTex, CA, catalog number: GTX629743), a mouse anti-human E-cadherin (1:1000; GeneTex, catalog number: GTX629691), a mouse anti-human CD24 (1:500; Novus Biologicals, Littleton, CO, USA, catalog number: NBP2-37352), a mouse anti-human CD26 (1:500; Novus Biologicals, catalog number: NBP2-02154), and a mouse anti-human CD133 (1:500; Novus Biologicals, catalog number: NBP2-52473). Secondary antibodies used were HRP-conjugated goat anti-mouse IgG (1:1000; Cell Signaling Technology, Beverly, MA, USA).

**Figure legends**

**Supplemental Figure S1. Transduction of *OCT3/4, KLF4 and SOX2* in MG-63 cells**

**A**: A schematic representation of a retroviral polycistronic vector and 2A-linked fusion gene (pMXs-OKS). Three defined transcription factors (*OCT3/4*, *KLF4* and *SOX2*) were fused in frame via 2A sequences and coexpressed as a single ORF. **B**: The transduction efficiency was assessed by GFP expression in MG-63 cells. Scale bars represent 100 µm. **C**: qRT-PCR of *OCT3/4*, *KLF4* and *SOX2* in transduced MG-63 cells. The mRNA expression levels were normalized to those of *β-*actin, and the expression level of MG-parental cells was set to 1. The error bars indicate the standard deviation: SD. *P<0.05, **P<0.02. **D**: Immunoblot analysis of CD24, CD26 and CD133 in transduced MG-63 cells.

**Supplemental Figure S2. The transduction of OKS induced CSC properties in NOS-1 cells *in vitro*.**

**A**: qRT-PCR of previously reported markers (*CD24*, *CD26* and *CD133*) related to CSCs of various cancers in the transduced NOS-1 cells. The mRNA expression levels were normalized to those of *β-*actin, and the expression level of NOS-parental cells was set to 1. The error bars indicate the standard error of the mean: SEM. *P<0.05. n.s: not significant. **B**: The morphology of the transduced NOS-1 cells under a phase contrast microscopy. The transduction of *OCT3/4*, *KLF4* and *SOX2* led to distinct morphological changes (arrow). Scale bars represent 20 µm.

**Supplemental Figure S3. Cell proliferation and migration assays in the transduced and parental NOS-1 cells *in vitro*.**

**A**: Cell proliferation was measured by WST-8 assay. **B**: Representative images of wound healing assays at 0 and 24 hours. **C**: The effects of transduction on the migration ability of NOS-1 cells. The migration distance (MD) in each group was calculated according to the following equation: MD = the width of the scratch at 0 hours - the width of the scratch at 24 hours. The MD value of the NOS-parental population was used as a reference. The relative cell migration ability was determined by the following equation: Relative cell migration ability = MD (NOS-OKS) or MD (NOS-GFP) / MD (NOS-parental). **D**: Doxorubicin-chemoresistance analysis. The viability of NOS-OKS cells in the presence of doxorubicin was evaluated by WST-8 assay. The viability of the NOS-parental cells at each concentration was set to 1. **E**: mRNA level of *ABCB1* was assessed by qPCR. The mRNA expression levels were normalized to those of *β-*actin, and the mRNA expression level of NOS-parental cells was set to 1. The error bars indicate the standard error of the mean: SEM. *P<0.05

**Supplemental Figure S4. Gene expression microarray analysis of the transduced and parental MG-63 cells**

**A**: Scatter plot showing the one-tenth downregulated genes (the blue dots). **B**: Venn diagram showing the number of the one-tenth downregulated genes in the comparison of MG-OKS vs MG-parental (left) and of MG-OKS vs MG-GFP (right), and the number of genes that are differential expression in both comparisons (center).

**References**

1 Yuan JS, Reed A, Chen F et al. Statistical analysis of real-time PCR data [in eng]. BMC bioinformatics 2006;7:85.

2 Shoji T, Ii M, Mifune Y et al. Local transplantation of human multipotent adipose-derived stem cells accelerates fracture healing via enhanced osteogenesis and angiogenesis [in eng]. Laboratory investigation; a journal of technical methods and pathology 2010;90(4):637-649.

3 Yuan Y, Zheng S, Li Q et al. Overexpression of miR-30a in lung adenocarcinoma A549 cell line inhibits migration and invasion via targeting EYA2 [in eng]. Acta Biochim Biophys Sin (Shanghai) 2016;48(3):220-228.

4 Xie Y, Sun W, Deng Z et al. MiR-302b Suppresses Osteosarcoma Cell Migration and Invasion by Targeting Runx2 [in eng]. Sci Rep 2017;7(1):13388.
