# Supplementary material for: Acquisition of cancer stem cell properties in osteosarcoma cells by defined factors
Source: Stem Cell Res Ther. 2020 Oct 2;11:429. doi: 10.1186/s13287-020-01944-9 (PMC7532109; doi:10.1186/s13287-020-01944-9)
Supplement: Supplementary file 3 — Additional file 3: Table S1. Primer sequences used in qRT-PCR. [file 13287_2020_1944_MOESM3_ESM.pdf]

Table S1. Primer sequences used in qRT-PCR.

Primer name	Sequence (5' to 3')		Size (bp)
hOCT3/4 (total)	Fwd	CCC CAG GGC CCC ATT TTG GTA CC	143
	Rev	ACC TCA GTT TGA ATG CAT GGG AGA GC	
hKLF4 (total)	Fwd	CAT GCC AGA GGA GCC CAA GCC AAA GAG GGG	132
	Rev	CGC AGG TGT GCC TTG AGA TGG GAA CTC TTT	
hSOX2 (total)	Fwd	TTC ACA TGT CCC AGC ACT ACC AGA	80
	Rev	TCA CAT GTG TGA GAG GGG CAG TGT GC	
CD24	Fwd	TGC TCC TAC CCA CGC AGA TT	89
	Rev	GGC CAA CCC AGA GTT GGA A	
CD26	Fwd	CAA ATT GAA GCA GCC AGA CA	133
	Rev	CAC ACT TGA ACA CGC CAC TT	
CD133	Fwd	TGG GGC TGC TGT TTA TTA TTC T	194
	Rev	TGC CAC AAA ACC ATA GAA GAT G	
ABCB1	Fwd	CGT GGT TGG AAG CTA ACC CT	153
	Rev	TGC TGC CAA GAC CTC TTC AG	
Osteocalcin	Fwd	ACA CTC CTC GCC CTA TTG	249
	Rev	GAT GTG GTC AGC CAA CTC	
BMP2	Fwd	ATG GAT TCG TGG TGG AAG TG	349
	Rev	GTG GAG TTC AGA TGA TCA GC	
BMP4	Fwd	AGC ATG TCA GGA TTA GCC GA	399
	Rev	TGG AGA TGG CAC TCA GTT CA	
BMP6	Fwd	CAG CCT GCA GGA AGC ATG AG	246
	Rev	CAA AGT AAA GAA CCG AGA TG	
β-actin	Fwd	GAT CAT TGC TCC TCC TGA GC	83
	Rev	ACA TCT GCT GGA AGG TGG AC	

hOCT3/4: human octamer-binding transcription factor 3/4 (OCT3/4); *hSOX2*: human SRY-box transcription factor 2 (SOX2); *hKLF4*: human Kruppel like factor 4 (KLF4); *ABCB1*: ATP-binding cassette sub-family B member 1; *BMP*: bone morphogenetic protein; Fwd: Forward; Rev: Reverse; bp: base pairs
